# The Good Examples Are Eternal

**DOI:** 10.5935/1678-9741.20150084

**Published:** 2015

**Authors:** Paulo Manuel Pêgo-Fernandes

**Affiliations:** 1 Full Professor and Chairman of Thoracic Surgery Division of the Instituto do Coração (InCor) da Universidade de São Paulo, SP, Brazil.

Writing about Prof. Adib Jatene is to propose the challenge of looking at a human being of unusual facets. Although I do not have the pretension to cover in this article the entire unique figure he was, I will take on this challenge as a tribute to the welcome opportunity I have had to live with him at the Heart Institute of the Clinics Hospital of the Faculty of Medicine of USP, for more than three decades.

Prof. Adib was a brilliant thinker and a worker and tireless innovator in search for answers to crucial questions of the society of his time. Recognized as a scientist, surgeon and admirable teacher, he was also a bold public manager (as Secretary of State for Health Department and Minister of Health) and head of an exemplary family.

I dare opine that all these qualities have the source in one of his most outstanding and brilliant features: the ever jovial look at the things in life (he invariably discovered something unusual where most saw only the obvious) and passion for the human being.

Yes, the compassionate look at his fellows made Prof. Adib Jatene example of an intellectual elite that has a real commitment to the country and its citizens.

His tireless efforts and his pursuit of perfection, possible in all his actions were nothing more than his aspiration, always there to help people who suffer to feel better.

There were many times when, in reflective conversations, Prof. Jatene cited Mother Teresa of Calcutta's quote to explain the flame that should guide the intelligence in the search for solution of the most different aspects of human life: "Without faith there is no love, without love there is no self-giving, and who is not able to make such giving is not prepared to deal with suffering."

Only full delivery to the common good is able to explain the unusual trajectory of Professor, from his childhood in Xapuri, Acre - where he was born on June 4, 1929, and also where he lost his father, yet when he was two years old, victim of fulminant disease acquired in the forest.

After all, it would not be unusual a fatherless young from the corners of the country, to follow the path of so many other millions of Brazilians who, while valuable in their daily struggle for survival, remain anonymous. But the story of this man who was guided by the passion for knowledge and the human being followed different path.

There were many of his titles and society representatives: Chairman of the Department of Cardiovascular the Brazilian Society of Cardiology Surgery (1985), "Honorary Member" of the American Association for Thoracic Surgery (1984), founding partner and president of the Brazilian Society of Cardiovascular Surgery (1984/1985), president of the Brazilian Society of Cardiology (1985/1987), president of the International Society for Cardiovascular Surgery (1985/1987).

He was also a member of the Committee of Experts of the Medical Education of the Ministry of Education (1986/1990), the National Health Council (1986/1992) and the Regional Council of Medicine of São Paulo (1988-1992). In 1989, he was elected full member of the National Academy of Medicine, and in October 1990, director of the School of Medicine at the University of São Paulo for four years.

He was a member of the National Council of Social Security and the Federal Ministry of Education and Minister of State for Health, twice.

In public administration, he introduced criticism in Accounts Processing System; implemented the Integrad Programming; created the Basic Care Package (PAB); chaired the 9^th^ and 10^th^ National Health Conference, drafted the Basic Operational Norm 1/96, which consolidated the National Health System, and gave great emphasis on programs of Family Health and Community Agents.

He fought for linking of resources and negotiated in record time with the World Bank and the Inter-American Development Bank, the Reforsus program, whose funds were distributed to several states according to their population.

It was the author and co-author of about 700 scientific papers published in national and international literature and a member of 32 scientific societies in many parts of the world. He received 178 titles and honors in more than ten countries.

Among its several unique contributions in the field of bioengineering, these include the bubble oxygenators and membrane and the tilting disc valve, of which he has the patent. These equipments are being industrially produced under license and used in the country and abroad.

Prof. Jatene still had important contributions in the field of coronary artery bypass graft surgery and surgery for congenital heart defects.

These feats earned him in 2007, in Greece, an award that much honored him: the "Seven Wise Men of the World in Cardiovascular Surgery", which awarded the luminaries of world Medicine for his contributions of unequivocal importance.

Indeed, his great joy with this award was not due to arrogance, but because it was an indication that his creations have resulted in great benefit to humanity. Prof. Adib described the ingenious technique of correction of transposition of the great arteries - known today as Jatene's operation, which has been successfully employed in several cardiac surgery centers worldwide.

The surgical teams he led, since 1962, performed more than 80,000 operations. Various services in the country and in South America are led by surgeons trained under his guidance. Moreover, in education he was a staunch defender of the quality of medical courses.

Doctor, family man, friend, scientist, innovative leadership, citizen and public exemplary man.

So, that was Prof. Adib.

Was?

No. He is still, such a good example is eternal. Prof. Adib Jatene remains a great inspiration for all of us and for future generations.

